# Successful Treatment of Aspergilloma With Antifungal Alone: A Case of Conservative Management

**DOI:** 10.1002/rcr2.70334

**Published:** 2025-08-27

**Authors:** Ad Rian Chong, Khai Lip Ng, Nai‐Chien Huan, Nur Husna Mohd Aminudin, Raja Nor Adilla Raja Rahaizat, Kasuma Mohamed Nordin

**Affiliations:** ^1^ Division of Respiratory Medicine, Department of Internal Medicine Melaka Hospital Melaka Malaysia; ^2^ Department of Respiratory Medicine Queen Elizabeth Hospital Kota Kinabalu Sabah Malaysia; ^3^ Centre for Innovative Pleural Research, Sir Charles Gairdner Hospital Perth Australia; ^4^ School of Medical and Health Sciences Edith Cowan University Perth Australia; ^5^ Department of Radiology Melaka Hospital Melaka Malaysia

**Keywords:** antifungal therapy, haemoptysis, isavuconazole, non‐surgical management, pulmonary aspergilloma

## Abstract

Pulmonary aspergilloma can cause life‐threatening haemoptysis. Surgical resection and/or bronchial artery embolization (BAE) are established treatment modalities, but both can be risky in frail patients with comorbidities. Spontaneous regression of aspergilloma with antifungal agents alone is rare. We report an elderly gentleman with a background history of treated pulmonary tuberculosis, who presented with haemoptysis due to a large left upper lobe aspergilloma. He declined surgery and BAE due to perceived risks. Oral voriconazole and later isavuconazole were prescribed, which led to clinical improvement and significant reduction in the size of the aspergilloma over 6 months. Antifungal agents might be a viable therapeutic option for aspergilloma patients unsuitable or who declined more invasive procedures. Further studies are needed to validate the efficacy and safety of this approach.

## Graphical abstract

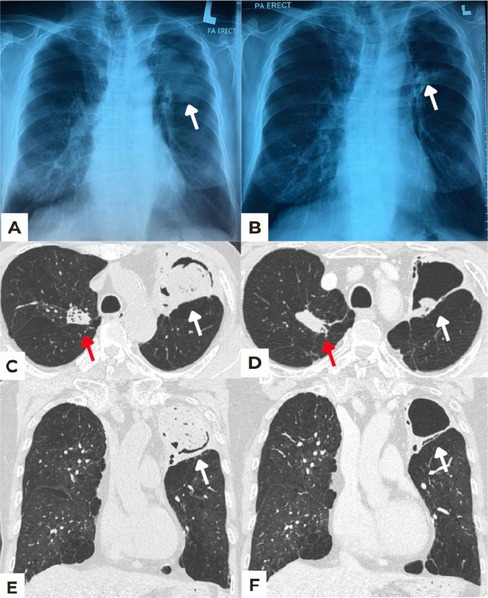

## 1 Introduction

Pulmonary aspergilloma, commonly termed fungal ball, is a non‐invasive form of pulmonary aspergillosis. It typically results from saprophytic fungal colonisation of pre‐existing lung cavities secondary to conditions such as tuberculosis and sarcoidosis. These fungal balls are composed of *Aspergillus* hyphae, conidia, necrotic debris, and mucus, and are most often due to *Aspergillus fumigatus*, although other species may be implicated [1]. Clinically, patients can be asymptomatic, present with chronic cough, or even life‐threatening haemoptysis. Surgical resection and/or bronchial artery embolization (BAE) are established treatment modalities for pulmonary aspergillomas [2].

Spontaneous resolution or regression of aspergillomas with anti‐fungal agents alone is rare. Anti‐fungal agents are generally regarded as disease‐stabilising rather than curative for aspergillomas because the avascular, necrotic environment of aspergillomas limits drug penetration. Herein, we report an elderly gentleman with marked reduction in aspergilloma size following anti‐fungal therapy, highlighting an unusual but clinically relevant outcome.

## 2 Case Report

A 76‐year‐old gentleman presented with haemoptysis, cough, and shortness of breath for the past 1 year. He was treated for pulmonary tuberculosis 40 years ago, which was complicated by cystic bronchiectasis and a left upper lobe lung cavity. He also underwent a right hemicolectomy in 2017 for stage three splenic flexure colon adenocarcinoma, with no evidence of recurrence or metastases on yearly surveillance.

**FIGURE 1:**
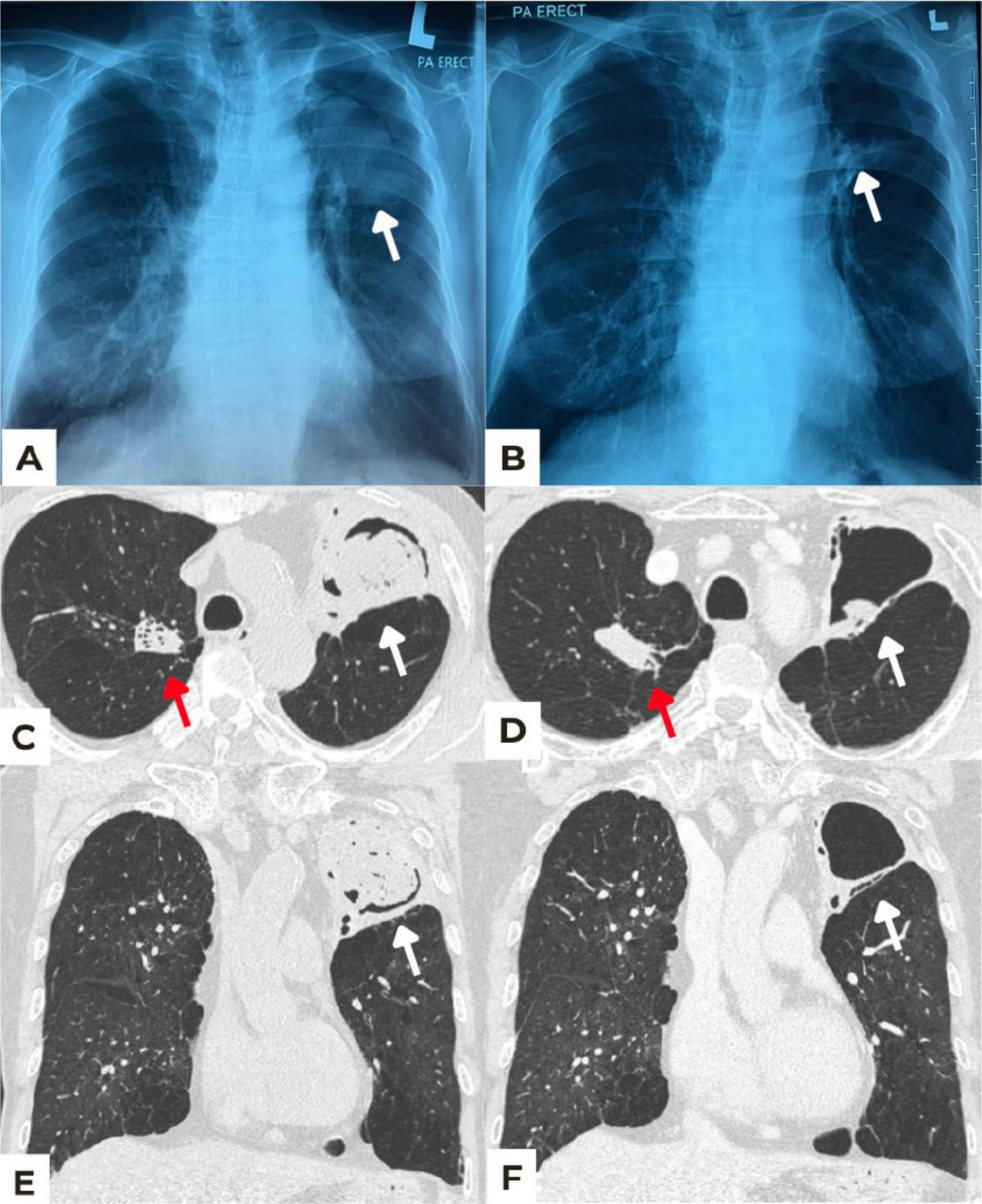
(A) Chest radiograph (PA erect view) demonstrates a well‐defined ovoid opacity with rim of lucent air at its periphery (white arrow), exhibiting an air crescent sign—a classic feature of a fungal ball (aspergilloma) in the left upper zone. (B) Follow‐up chest radiograph (PA erect view) post‐treatment shows marked reduction in the size of the aspergilloma (white arrow), accompanied by increased aeration of the left upper zone cavity, indicating a favourable radiological response to therapy. (C) Axial contrast‐enhanced CT thorax showing thin‐walled cavity in the left upper lobe containing an intracavitary soft‐tissue lesion consistent with a fungal ball (mycetoma) (white arrow) measuring approximately 4.5 × 6.0 × 5.7 cm. There is right apical fibrosis (red arrow) and background emphysematous lung. (D) Follow‐up CT thorax post‐treatment shows significant reduction in the size of the fungal ball within the pre‐existing left upper lobe cavity (white arrow), now measuring approximately 2.6 × 1.2 × 2.2 cm. Persistent right apical fibrosis is noted (red arrow). (E) Coronal reformatted image of C demonstrating the same cavity and fungal ball. (F) Coronal reformatted image of D demonstrating interval reduction in the size of the fungal ball within the left upper lobe cavity.

Physical examination was unremarkable, but chest radiograph and computed tomography (CT) scan of the thorax, abdomen, and pelvis revealed a large aspergilloma within the left upper lobe lung, measuring 4.5 × 6.0 × 5.7 cm (Figure 1). Bronchoscopy was performed and his bronchoalveolar lavage (BAL) galactomannan, cultures, and polymerase chain reaction (PCR) all returned positive for *Aspergillus fumigatus*. A follow‐up CT angiogram was conducted due to persistent haemoptysis, which showed focal dilatation of a branch of the left descending pulmonary artery traversing the cavity, raising suspicion of Rasmussen aneurysm.

He was offered surgery or BAE but declined due to perceived risks: advanced age, concerns over potential complications such as bleeding or spinal cord ischaemia, and an anticipated long recovery period post‐surgery. Oral voriconazole was therefore initiated, which led to resolution of haemoptysis. Voriconazole was withheld after a week due to derangements in liver enzymes. Ultrasound of the hepatobiliary system showed cholelithiasis without biliary obstruction, and viral hepatitis screenings were negative. Following discussion, oral isavuconazole was initiated—at a loading dose of 200 mg thrice per day for the first two days, followed by a once‐daily 200 mg maintenance dose. His liver function remained normal on isavuconazole.

He reported multiple episodes of coughing up blackish materials during clinic follow‐ups, presumed to be necrotic fungal debris. He subsequently completed a total of 6 months of isavuconazole therapy, which was associated with the resolution of respiratory symptoms. A repeated CT thorax after 6 months of treatment showed a significant reduction in the size of the aspergilloma to 2.6 × 1.2 × 2.2 cm.

## 3 Discussion

Spontaneous regression or resolution of pulmonary aspergilloma is rare—as low as 5% from various sources [3]. The exact pathophysiology of this phenomenon remains unclear. Postulates include aspergilloma cavity collapse, fibrosis and scarring leading to involution, spontaneous expectoration of fungal materials, or anti‐fungal agent induced cavity degradation. Anti‐fungal therapy may reduce the total body fungal burden and facilitate spontaneous clearance in a minority of patients [4]. In our case, our patient reported coughing out blackish materials, presumed to represent fungal materials. This may have contributed to the reduction in aspergilloma size, but it remains unclear whether this event was spontaneous or facilitated by anti‐fungal agents. Predictors of spontaneous aspergilloma resolution are not known. Factors such as cavity wall thickness, anatomical location, lesion chronicity, and patients' immune status may be implicated, but lack validation.

Managing pulmonary aspergillomas can be challenging. Surgical resection and/or BAE are well‐established treatment modalities—but both are associated with potential complications. Traditional surgical approaches such as posterolateral thoracotomy can be complicated due to chest wall adhesions and the inflammatory nature of the condition, with reported morbidity and mortality of up to 33.3% and 3.3%, respectively [2]. Video‐assisted thoracoscopic surgery (VATS) is a less invasive alternative, potentially reducing complications. Nonetheless, many patients with pulmonary aspergillomas have poor cardio‐respiratory reserves due to underlying chronic conditions, rendering surgical resection prohibitively risky.

BAE is useful for patients experiencing haemoptysis, particularly for those unsuitable for surgery. It is effective in 50%–90% of cases, improving quality of life by controlling airway bleeding [5]. Recurrence rates, however, can be high, ranging from 19% to 55%, necessitating repeated procedures or concurrent antifungal therapy. Non‐target embolization leading to unintentional ischaemia in other organs such as the spinal cord is a rare but significant complication of BAE [6].

In patients deemed unsuitable or who declined invasive therapies, anti‐fungal agents such as voriconazole, itraconazole, and amphotericin‐B have been used with varying degrees of success [7]. Voriconazole is the preferred agent for *Aspergillus fumigatus* , but its use can be limited by hepatotoxicity, visual disturbances, and skin reactions [1]. Intra‐cavitary amphotericin‐B can be effective in patients with acute haemoptysis—but significant toxicities can be a limiting factor [8]. Isavuconazole is a newer generation triazole with broad‐spectrum activity, a more favourable safety profile, fewer drug interactions, and less need for routine therapeutic drug monitoring [9]. It has emerged as a viable alternative in the management of chronic pulmonary aspergillosis. De Oliveira et al. reported a case series of 10 patients with chronic pulmonary aspergillosis who were successfully treated with isavuconazole as salvage therapy. Most patients in the series were intolerant to voriconazole or itraconazole—like our case [10].

The decision to pursue conservative management of pulmonary aspergillomas should be individualised. It must tactfully consider patient characteristics with a careful balance of benefits against potentially fatal complications, especially massive haemoptysis. No consensus exists on the optimal antifungal choice and duration. Isavuconazole's role is encouraging, but larger studies are needed to validate its efficacy and safety.

In conclusion, this case report suggests isavuconazole as a potentially viable therapeutic option for managing pulmonary aspergilloma in patients unsuitable for invasive procedures. Further studies are needed to determine its optimal dosage and duration, as well as to compare its efficacy and safety with other antifungal agents.

## Author Contributions

Ad Rian Chong, Khai Lip Ng, Nai‐Chien Huan contributed to the design and implementation of the case report. Ad Rian Chong, Khai Lip Ng, Nai‐Chien Huan wrote the manuscript. Ad Rian Chong, Khai Lip Ng, and Nur Husna Mohd Aminudin carried out the procedure and treatment mentioned. Raja Nor Adilla Raja Rahaizat reviewed all the radiological images and interpreted the radiological findings. Kasuma Mohamed Nordin supervised the project. All authors discussed the study and contributed to the final manuscript.

## Ethics Statement

The authors declare that written informed consent was obtained for the publication of this manuscript and accompanying images using the form provided by the Journal.

## Conflicts of Interest

The authors declare no conflicts of interest.

## Data Availability

Data sharing not applicable to this article as no datasets were generated or analysed during the current study.
